# Is Exposure to Certain Endocrine-Disrupting Chemicals Associated with Increased Skin Cancer Risk?

**DOI:** 10.3390/cancers18203283

**Published:** 2026-10-12

**Authors:** Grigoria Betsi, Dimitra Koumaki, Anastasia Kountouri, Paraskevi Xekouki

**Affiliations:** 1 Department of Endocrinology and Diabetes, University Hospital of Heraklion, 71110 Crete, Greece; gbetsi@pagni.gr (G.B.); pxekouki@uoc.gr (P.X.); 2 2nd Department of Dermatology and Venereology, Medical School, “Attikon” General University Hospital, National and Kapodistrian University of Athens, Rimini 1, Haidari, 12461 Athens, Greece

**Keywords:** endocrine disruptors, PCB, PAH, PFAS, dioxin, pesticides, arsenic, skin cancer, melanoma

## Abstract

Background/Objectives: This review investigated whether exposure to certain endocrine-disrupting chemicals (EDCs) increases skin cancer risk. Methods: We performed a comprehensive narrative review of in vitro, animal, and human studies examining the association of EDCs with melanoma and non-melanoma skin cancer (NMSC). Results: Chemicals are classified as (i) established EDCs with evidence of skin carcinogenicity; (ii) chemicals that are carcinogenic with endocrine-related effects; and (iii) chemicals with limited or uncertain evidence. Proposed carcinogenic mechanisms include oxidative stress, chronic inflammation, immunosuppression, DNA repair inhibition, and epigenetic alterations; AhR likely mediates the carcinogenicity of dioxin (TCDD), dioxin-like PCBs, and PAHs. Epidemiological evidence linking PCBs with melanoma is inconsistent. High cumulative occupational exposure to PAH-containing mineral oils or tar/pitch increases skin cancer risk. Evidence for PFAS and skin cancer is limited and inconsistent. Increased melanoma incidence has been reported after chronic occupational exposure to certain pesticides, including carbaryl, parathion, and maneb/mancozeb. There is substantial evidence of a dose–response association between chronic arsenic exposure and NMSC risk. Conclusions: High chronic exposure to specific EDCs (especially arsenic, certain PAHs, and certain pesticides) may increase skin cancer risk. For several chemicals, evidence supports carcinogenicity with endocrine-related effects, but direct mechanistic evidence that endocrine disruption specifically mediates skin carcinogenesis is lacking.

## Simple Summary

This narrative review investigates whether environmental factors beyond sun exposure contribute to skin cancer development. Since skin expresses hormone receptors involved in cell proliferation, immunity, and melanogenesis, exposure to endocrine-disrupting chemicals (EDCs) may disturb skin homeostasis and contribute to carcinogenesis. Identifying modifiable EDC-related risk factors may also provide insights for hormone-based treatments.

## 1. Introduction

The US EPA defines an environmental endocrine disruptor as “an exogenous agent that interferes with the synthesis, secretion, transport, binding, action, or elimination of natural hormones in the body responsible for the maintenance of homeostasis, reproduction, development and/or behavior” [1]. The WHO defines an EDC as “an exogenous substance or mixture that alters function(s) of the endocrine system and consequently causes adverse health effects in an intact organism, or its progeny, or (sub)populations” [2].

Endocrine-disrupting chemicals (EDCs) can negatively affect reproduction, thyroid function, and metabolism and may impact pituitary and adrenal function [3]. Exposure occurs through contaminated food or water, air inhalation, or skin contact; although the skin functions as a barrier, some chemicals penetrate the epidermis and interfere with hormones. Some EDCs have been associated with skin diseases including contact dermatitis, chloracne, and hyperkeratosis [4].

Throughout this review, we distinguish between (i) evidence that a compound has endocrine-disrupting activity; (ii) evidence of an association with skin cancer; and (iii) evidence, where available, that endocrine signalling mechanistically links the two. Many proposed mechanisms of skin carcinogenesis (DNA damage, inhibition of DNA repair, oxidative stress, epigenetic alterations) do not in themselves demonstrate endocrine-mediated carcinogenesis.

## 2. Methods

This is a comprehensive narrative review. A structured literature search was conducted in PubMed/MEDLINE and Scopus, last updated in August 2026. Search terms combined “endocrine disrupting chemicals”, “skin cancer”, “melanoma”, “non-melanoma skin cancer”, “basal cell carcinoma”, and “squamous cell carcinoma” with individual chemical classes. No language restriction was applied. Original studies and meta-analyses on EDC exposure and skin cancer incidence, mortality, or risk were included; case reports and editorials were excluded.

EDC classes were selected based on (a) classification as EDCs by international regulatory agencies; (b) the availability of human epidemiological data on skin cancer outcomes; and (c) sufficient mechanistic or experimental evidence.

The principal proposed mechanisms of EDC-associated skin carcinogenesis are summarised in Table 1.

**Evidence grading definitions**

- *“Sufficient evidence”*: consistent findings across multiple independent epidemiological studies with dose–response data.
- *“Substantial evidence”*: broadly concordant findings from multiple study types (in vitro, animal, and human).
- *“Moderate evidence”*: some epidemiological data with inconsistent results or methodological limitations.
- *“Limited or inconclusive evidence”*: sparse or conflicting human data, primarily from experimental models.

Chemicals are classified into three categories:

1. Established EDCs with evidence of skin carcinogenicity (e.g., arsenic, certain PAHs, certain pesticides).
2. Chemicals that are carcinogenic with endocrine-related effects (e.g., PCBs, TCDD, trichloroethylene).
3. Chemicals with limited or uncertain evidence (e.g., PFAS, PBDEs, phthalates, parabens).

## 3. Expression of Hormone Receptors in Skin Cancer Cells

### 3.1. Melanoma

Malignant melanomas predominantly express oestrogen receptor (ER) β, while ERα is usually decreased or absent [5]; significantly lower ERβ expression has been detected in melanoma compared to adjacent healthy skin [6]. G-protein-coupled oestrogen receptor (GPER) is expressed in human melanoma cells and induces melanogenesis via the upregulation of tyrosinase, MITF, and the cAMP-PKA pathway [7]. Androgen receptor (AR) is expressed variably in melanoma cells and can induce invasiveness through MITF downregulation, epithelial-to-mesenchymal transition (EMT), and MMP upregulation [8]. TSH receptors are expressed in all melanocytic lesions, with greater expression in malignant melanomas, promoting cAMP production and MAPK pathway activation [9]. Melanoma cells also express GH, insulin, and IGF receptors [10]; IGF1 and insulin activate MAPK, Akt/β-catenin, and PI3K/Akt signalling. Most melanoma cells express high cytosolic glucocorticoid receptor (GR) levels [11].

### 3.2. Keratinocyte Cancers (Basal Cell Carcinoma and Squamous Cell Carcinoma)

Keratinocytes express functionally relevant hormone receptors. ERβ is predominantly expressed in normal keratinocytes and in BCC and SCC tissue, potentially exerting antiproliferative effects. AR is expressed in sebaceous glands and hair follicles and may modulate keratinocyte proliferation. The aryl hydrocarbon receptor (AhR), the primary mediator of dioxin and PAH toxicity, is highly expressed in keratinocytes and plays a direct role in skin homeostasis, UV response, and the pathogenesis of both BCC and SCC. The activation of AhR in keratinocytes by environmental ligands (TCDD, PAHs) induces CYP1A1 and CYP1B1, generating reactive metabolites capable of DNA adduct formation, providing a biologically plausible basis for EDC-associated NMSC risk.

## 4. Polychlorinated Biphenyls (PCBs) and Skin Cancer

PCBs were widely used in electrical capacitor plants and persist as environmental pollutants. Exposure occurs via skin contact, digestion, or inhalation.

### 4.1. PCBs as EDCs

Lower-chlorinated PCB congeners and hydroxylated metabolites can act as oestrogen agonists, with binding affinities to ERα and ERβ only 20- to 40-fold lower than for E2, while many higher-chlorinated congeners are anti-oestrogenic [12]. PCBs can also act as anti-androgens [13,14], and some PCB mixtures displace dihydrotestosterone from AR binding [15]. Plasma PCB levels have been negatively associated with testosterone [16]. Some higher-chlorinated PCBs may act as thyroid receptor (TR) antagonists, while some lower-chlorinated congeners may act as TR agonists [17,18].

### 4.2. Carcinogenicity of PCBs

PCBs are classified by the IARC as Group 1 carcinogens. According to the IARC Working Group, evidence in humans is sufficient to conclude that PCBs may cause melanoma [19]. Potential mechanisms include genotoxicity, metabolic activation to mutagenic PCB quinones, altered DNA repair, epigenetic alterations, oxidative stress, chronic inflammation, immunosuppression, and apoptosis inhibition [19]. Many carcinogenic effects of dioxin-like PCBs are mediated through AhR, a cytosolic transcription factor expressed on human melanocytes [20], which, upon ligand binding, regulates the transcription of CYP1A1 and CYP1B1, promoting the production of DNA-reactive metabolites and pro-inflammatory gene expression [21]. AhR-mediated mechanisms represent the most plausible link between the endocrine-disrupting and carcinogenic properties of dioxin-like PCBs, as AhR cross-talks with oestrogen and androgen receptor signalling.

### 4.3. In Vitro Studies of Skin Carcinogenicity of PCBs

Some airborne PCB congeners (e.g., PCB28, PCB52) may reduce telomerase activity and shorten telomeres in human keratinocytes, potentially causing genomic instability [22]. Older studies failed to demonstrate the carcinogenicity of technical PCB mixtures in mice [23]. The experimental concentrations in many in vitro studies substantially exceed typical human exposure levels, limiting direct extrapolation.

### 4.4. Human Studies of Skin Carcinogenicity of PCBs

The epidemiological evidence is summarised in Table 2a. Studies focus primarily on occupational cohorts with co-exposures and variable UV exposure.

A case–control study of 80 melanoma patients and 310 controls found strong associations with total summed (OR 6.02) and non-dioxin-like (OR 7.02) plasma PCB levels, adjusting for sun exposure [24]. A follow-up in 406 melanoma cases found a significantly higher melanoma risk with higher plasma concentrations of non-dioxin-like PCB congeners [25]. A Chinese case–control study (450 melanoma/500 controls) found a significantly increased risk in the highest PCB quartile (OR 1.44) [26]. Conversely, a Northern Italian case–control study found no significant association [27].

In studies with concurrent exposures, a cohort of 92 German transformer-recycling workers with elevated PCB/dioxin levels showed increased melanoma, mycosis fungoides, and BCC incidence (NMSC period-adjusted incidence rate ratio 5.6, 95% CI 2.6–12) [28]. Excess melanoma mortality was reported among US electrical power company workers [29] and capacitor-manufacturing plant workers [30] and among Norwegian hydroelectric workers, with a dose–response relationship (melanoma and NMSC incidence rose significantly after >30 years of employment) [31].

No excess melanoma mortality was detected in a 50-year follow-up of Yusho patients who had ingested PCB/PCDF-contaminated rice bran oil [32] or in several other cohorts [33,34,35,36].

A meta-analysis of 12 studies found no significant association (random-effect RR 1.05) [37]. A meta-analysis of 11 occupational cohorts found a moderate increase in the cutaneous melanoma mortality risk (pooled SMR 1.32, 95% CI 1.05–1.64), with inconsistent dose–response results [38]. A more recent meta-analysis found significantly increased melanoma mortality in chemical industry workers possibly exposed to PCBs (eight cohort studies, SMR 2.01, 95% CI 1.09–3.72) but no increase in electrical industry workers [39].

## 5. Dioxin and Skin Cancer

2,3,7,8-Tetrachlorodibenzo-p-dioxin (TCDD) is both an endocrine disruptor and a Group 1 IARC carcinogen.

### 5.1. Dioxin/TCDD as an EDC

Dioxin may disturb reproductive, thyroid, and endocrine pancreatic function. Among Air Force veterans of Operation Ranch Hand, high dioxin exposure was significantly associated with insulin-requiring diabetes and higher mean TSH levels [40].

### 5.2. Mechanistic and Experimental Evidence

TCDD is not a mutagen. AhR mediates dioxin immunotoxicity and carcinogenicity [40]: TCDD activates AhR in normal melanocytes, upregulating CYP1A1, CYP1B1, and tyrosinase [20], and, in invasive melanoma cells, it induces MMPs, increasing invasiveness [41]. In animal studies, a dose-dependent increase in cutaneous squamous cell papillomas and SCC was observed in female mice after dermal or oral TCDD [42].

### 5.3. Human Studies of TCDD Exposure and Skin Cancer

The epidemiological evidence is summarised in Table 2b. Most studies are occupational or veteran cohorts and did not explicitly adjust for UV exposure. A study of US Air Force veterans found an increased melanoma incidence and a suggested association with dioxin exposure [43], and a retrospective study found a significantly higher invasive NMSC incidence (51%) in 100 TCDD-exposed Vietnam veterans compared to age-matched US controls [44]. The Air Force Health Study (AFHS) found a significantly higher prevalence of malignant skin neoplasms (especially BCC) in personnel with low dioxin exposure but no significant association between melanoma or SCC and dioxin exposure [40,45]. Studies of Seveso residents, US workers, and multinational phenoxy herbicide cohorts found no significant increase in skin cancer risk [46,47,48].

## 6. Trichloroethylene and Skin Cancer

Trichloroethylene is a chlorinated solvent classified by the IARC as a Group 1 carcinogen based on sufficient evidence for kidney cancer [49]. Trichloroethylene and its metabolites may act as endocrine disruptors; chronic occupational exposure was associated with altered testosterone, FSH, SHBG, DHEAS, and insulin levels [50].

Evidence for skin cancer is limited and inconsistent. A Swedish cohort showed a doubled NMSC incidence in exposed workers [51]. Canadian case–control studies found a significantly increased melanoma risk with trichloroethylene exposure (OR 3.0–3.6) [52,53], and increased melanoma mortality was found among US personnel exposed to contaminated drinking water [54]. Conversely, several cohort studies found no association [55,56].

## 7. Polybrominated Diphenyl Ethers (PBDEs) and Polybrominated Biphenyls (PBBs)

Lower-brominated PBDEs have been associated with altered thyroid hormone levels; some hydroxylated congeners may weakly activate ERα and ERβ [21]. PBBs did not promote skin tumour development in mice [23]. An in vitro study found that PBDEs increased murine melanoma cell migration and overexpressed MMP2, MMP9, and Tp53 [57]. A case–control study of a Michigan population accidentally exposed to PBBs found no excess melanoma risk [58]. Overall, the evidence is insufficient to establish an association between PBB/PBDE exposure and skin cancer in humans.

## 8. Pesticides and Skin Cancer

Many pesticides act as EDCs; numerous organochlorines, such as DDT, have oestrogenic properties, while others antagonise ERα, ERβ, or androgen receptors [59]. In the Agricultural Health Study (AHS), the use of several pesticides was associated with an increased risk of hypothyroidism [60].

### 8.1. Mechanisms of Pesticide Carcinogenesis

Proposed mechanisms include the upregulation of gene expression/mutations (K-ras, p53, CYP, Cyclin D1), DNA repair inhibition, epigenetic alterations, apoptosis dysregulation, oxidative stress, and telomere shortening [61,62]. In a mouse skin carcinogenesis model, glyphosate showed tumour-promoting (but not tumour-initiating) activity on DMBA-initiated skin [63].

### 8.2. Human Studies of Pesticide Exposure and Skin Cancer

The epidemiological evidence is summarised in Table 2b. Studies are heterogeneous; most populations had significant outdoor UV exposure, and few formally adjusted for UV or phototype.

Several cohort and case–control studies suggest an increased skin cancer risk with pesticide exposure, including Italian licensed pesticide users (SIR 1.4) [64], male Swedish veterinary workers (SIR 2.86) [65], the AGRICAN cohort of French farm workers (melanoma in women, SIR 1.23) [66], and a Canadian case–control study finding increased SCC with insecticide, herbicide, and fungicide exposure [67].

An increased melanoma risk has been reported after prolonged (usually >10 years) exposure. An Italian case–control study found excess melanoma among female farmers (OR 2.7), being highest with 10–19 years of employment (OR 6.6) [68]. A pooled Italian–Brazilian analysis showed an approximately sevenfold higher melanoma risk with ≥10 years of exposure (OR 7.40) and fourfold higher with ≥2 pesticide types [69]. The AHS found an over threefold melanoma risk with the highest lifetime carbaryl use (RR 3.55–4.11) [70] and significant dose–response associations for carbaryl (≥56 lifetime days: OR 1.7, 95% CI 1.1–2.5), maneb/mancozeb (≥63 days: OR 2.4, 95% CI 1.2–4.9), and parathion (≥56 days: OR 2.4, 95% CI 1.3–4.4) [71]. A French–West Indies study found a 93% excess melanoma risk in men with moderate occupational chlordecone exposure (RR 1.93) [72]. A borderline significant association between acetochlor use and melanoma (RR 2.55, 95% CI 1.45–4.48 after UV adjustment) was also found in the AHS [73].

However, many studies found no increased skin cancer risk [74,75,76]. An international AGRICOH consortium found an increased melanoma risk among female agricultural workers (meta-SIR 1.18, 95% CI 1.01–1.38) [77]. A meta-analysis found a significant increase in melanoma risk related to herbicide use (SRR 1.85, 95% CI 1.01–3.36) without between-study heterogeneity, with no significant association for insecticide or overall pesticide exposure [78].

Overall, the evidence supports an association between prolonged occupational exposure to specific pesticides (particularly carbaryl, parathion, maneb/mancozeb) and melanoma risk; class-level generalisations are not supported.

## 9. Polycyclic Aromatic Hydrocarbons (PAHs) and Skin Cancer

PAHs are formed during the incomplete combustion of organic matter and represent one of the strongest areas of evidence in this review, particularly for occupational exposures.

### 9.1. PAHs as Endocrine-Related Chemicals

PAHs have been associated with the disruption of oestrogen and androgen signalling [79]. The primary carcinogenic mechanism is AhR activation and DNA adduct formation.

### 9.2. Mechanisms of PAH Carcinogenesis

AhR mediates PAH carcinogenicity (e.g., benzo[a]pyrene); AhR knockout mice are resistant to benzo[a]pyrene-induced carcinogenesis [80]. Skin expresses CYP1A1 and CYP1B1, which metabolise PAHs to reactive carcinogenic metabolites [81].

### 9.3. Occupational PAH Exposure and Skin Cancer

There is sufficient evidence that high cumulative occupational exposure to PAH-containing mineral oils or tar/pitch increases skin cancer risk (particularly SCC and NMSC), as demonstrated across multiple industries: oil shale workers [82], metalworking/machining workers exposed to straight metalworking fluids [83,84], tar refinery workers [85], highway asphalt and road construction workers [86,87], chimney sweeps [88], coke plant workers [89], and aluminium and carbon electrode industry workers [90,91]. A meta-analysis of asphalt workers and roofers found an increased skin cancer risk [86], as did meta-analyses of petroleum industry workers [92,93]. For non-occupational environmental PAH exposure, evidence for skin cancer is much more limited.

## 10. Per- and Polyfluoroalkyl Substances (PFAS) and Skin Cancer

PFAS are highly persistent synthetic fluorinated compounds. Some PFAS can activate ERα and interact with oestrogen-regulated genes in vitro [94] and have been associated with genotoxic effects [95] and altered immune function and lipid metabolism [96].

Evidence for PFAS and skin cancer is limited and inconsistent. Studies near a PFOA-contaminated plant found a non-significant positive association with melanoma [97,98]; a cohort incidence study of PFOA-exposed workers found a non-significantly increased melanoma risk [99]. Studies of PFOS manufacturing facility workers [100,101] and a Swedish cohort with high PFAS levels in drinking water [102] found no significant increase in skin cancer risk. US NHANES cross-sectional studies produced inconsistent results [103,104]. A retrospective Italian study found that PFAS exposure was associated with worse melanoma prognostic features [105]. Overall, the evidence is insufficient to establish a causal association between PFAS exposure and skin cancer.

## 11. Arsenic and Skin Cancer

Arsenic is a naturally occurring metalloid found in contaminated drinking water, soil, and certain foods. Chronic arsenic exposure is an established cause of NMSC, and it is best classified as a chemical with substantial carcinogenic evidence and endocrine-related effects.

### 11.1. Arsenic as an EDC

Arsenic may disrupt gonadal, adrenal, and thyroid endocrine function [106].

### 11.2. Mechanisms of Arsenic Carcinogenesis

The IARC classifies arsenic as a Group 1 carcinogen [107]. Although not a direct mutagen, arsenic can induce DNA damage through the inhibition of DNA repair (particularly nucleotide excision repair) and induction of oxidative stress [108,109]. Additional mechanisms include epigenetic alterations, altered gene expression in keratinocytes [110], and co-carcinogenesis with UV radiation in animal models [111,112].

### 11.3. Human Studies of Arsenic Exposure and Skin Cancer

There is substantial evidence from ecologic and cohort studies of a dose–response association between chronic arsenic exposure and NMSC risk. Landmark Taiwanese studies demonstrated a linear dose–response relationship between the arsenic concentrations in well water and NMSC prevalence and mortality [113,114,115], which was confirmed in studies from the US [116], Europe [117,118,119,120], and Asia [121]. A meta-analysis confirmed a significant dose–response association for both BCC and SCC [122].

Evidence for arsenic and melanoma is more limited: a NHANES cross-sectional analysis found a significant association between urinary arsenic levels and melanoma [123], and a meta-analysis found a positive association between arsenic exposure and melanoma risk [124]; however, several studies found no association [125,126]. The evidence for a causal association between chronic arsenic exposure and NMSC is among the strongest of any chemical discussed in this review.

## 12. Other Chemicals

### 12.1. Metals

A population-based case–control study found a non-significant positive association between dietary cadmium intake and cutaneous melanoma risk [127]. A cross-sectional NHANES study found associations between exposure to arsenic, PAHs, and certain metals and skin cancer [128]. Studies of trace element levels in melanoma patients have produced inconsistent results [129,130,131].

### 12.2. Phthalates

DEHP and other phthalates are EDCs with established antiandrogenic properties [132]. In murine models, DEHP enhanced melanoma tumour growth via differential effects on macrophage polarisation [133] and stimulated skin pigmentation [134]. Human evidence specifically for skin cancer is insufficient to draw conclusions.

### 12.3. Polyvinyl Chloride (PVC) and Vinyl Chloride Monomer (VCM)

VCM is classified by the IARC as a Group 1 carcinogen based on sufficient evidence for angiosarcoma of the liver. Evidence for skin cancer is limited, with inconsistent results in studies of PVC processing workers [135,136,137].

### 12.4. Diethylstilboestrol (DES)

DES is a synthetic oestrogen. In vitro, DES enhances melanogenesis via the cAMP-PKA upregulation of tyrosinase and MITF [138]. A Dutch cohort study of DES-exposed daughters found no significantly increased melanoma risk [139].

### 12.5. Parabens

Parabens are preservatives with weak oestrogenic activity [140]. Methylparaben potentiates UV-induced damage to skin keratinocytes in vitro [141]. Human evidence linking paraben exposure to skin cancer is absent.

## 13. Summary of Mechanistic Evidence

The principal proposed mechanisms of EDC-associated skin carcinogenesis are summarized in Table 1.

**Table 1:** Principal proposed mechanisms of EDC-associated skin carcinogenesis, based on skin-specific experimental evidence.

Mechanism	Chemical(s)	Model	Notes
AhR activation → CYP1A1/CYP1B1 induction → DNA adducts	TCDD, dioxin-like PCBs, PAHs	Melanocytes, keratinocytes, in vivo mouse skin	Primary mechanism for dioxin-like compounds and PAHs
Oxidative stress → DNA damage	PCBs, arsenic, PAHs	Keratinocytes, in vitro	Relevant to both melanoma and NMSC
DNA repair inhibition	Arsenic	Keratinocytes, human studies	Nucleotide excision repair inhibition
Epigenetic alterations (DNA methylation)	PCBs, arsenic, pesticides	Keratinocytes, in vivo	—
Immunosuppression (IL-1β upregulation, T-cell suppression)	PCBs	Skin, in vivo	May promote tumour immune evasion
Telomere shortening	Airborne PCBs (PCB28, PCB52)	Human keratinocytes (HaCaT)	In vitro only; experimental concentrations may exceed human exposure levels
MMP upregulation → increased invasiveness	TCDD, PBDEs	Melanoma cells	—
Co-carcinogenesis with UV radiation	Arsenic, PAHs	Mouse skin, in vivo	Arsenic and UV act synergistically
Oestrogen receptor-mediated melanogenesis (GPER)	Oestrogenic EDCs (DES)	Melanocytes, melanoma cells	Indirect link via ER signalling

## 14. Summary of Human Epidemiological Evidence

The epidemiological evidence is summarised in Table 2a. The epidemiological evidence is summarized in Table 2b.

**Table 2:** (**a**). Key human studies: PCBs, dioxin/TCDD, and skin cancer. (**b**) Key human studies: PAHs, pesticides, arsenic, and other chemicals and skin cancer.

(**a**)							
**Reference**	**Agent**	**Study Design**	**Population**	**Cancer Endpoint**	**Effect Estimate (95% CI)**	**UV/Phototype Adjusted**	**Principal Limitation**
Gallagher et al., 2011 [24]	Total and non-dioxin-like PCBs (plasma)	Case–control	80 melanoma, 310 controls, Canada	Melanoma	OR 6.02 (total PCBs); OR 7.02 (non-dioxin-like)	Yes	Small sample
Darvishian et al., 2022 [25]	Non-dioxin-like PCBs (plasma)	Case–control	406 melanoma, 181 controls, Canada	Melanoma	Significant for PCBs 138, 170, 180, 183, 187	Yes	Selection bias possible
Cao et al., 2019 [26]	Total PCBs (plasma)	Case–control	450 melanoma, 500 controls, China	Melanoma	OR 1.44 (highest vs. lowest quartile)	Yes	Chinese population; generalisability uncertain
Magoni et al., 2018 [27]	Total PCBs (plasma)	Case–control	205 melanoma, 205 controls, Italy	Melanoma	OR 0.81 (0.34–1.96)	Yes	No significant association
Leijs et al., 2018 [28]	PCBs + dioxins	Cohort	92 transformer-recycling workers, Germany	Melanoma, BCC, NMSC	IRR NMSC 5.6 (2.6–12)	No	Co-exposures (dioxins, solvents)
Loomis et al., 1997 [29]	Cumulative PCBs	Cohort	138,905 US electrical power workers	Melanoma mortality	RR 4.81 (>10,000 h, 20-year lag)	No	Co-exposures (mineral oils, solvents)
Ruder et al., 2014 [30]	PCBs	Cohort	24,865 US capacitor workers	Melanoma mortality	SMR 1.41 (1.01–1.91)	No	No dose–response
Boffetta et al., 2018 [37]	PCBs	Meta-analysis	12 studies	Melanoma	Fixed-effect RR 0.91; random-effect RR 1.05	Variable	Heterogeneity across studies
Zani et al., 2017 [38]	PCBs (occupational)	Meta-analysis	11 cohort studies	Melanoma mortality	Pooled SMR 1.32 (1.05–1.64)	Variable	Inconsistent dose–response
Ketchum et al., 1999 [45]	TCDD (serum)	Cohort	AFHS Operation Ranch Hand veterans	Melanoma, SCC, BCC	No significant association	No	No UV adjustment; outdoor exposure likely
Clemens et al., 2014 [44]	TCDD (Agent Orange)	Retrospective cohort	100 Vietnam veterans	Invasive NMSC	51% vs. general population	Partial (skin type)	Small sample; no control group
(**b**)							
**Reference**	**Agent**	**Study Design**	**Population**	**Cancer Endpoint**	**Effect Estimate (95% CI)**	**UV/Phototype Adjusted**	**Principal Limitation**
Costello et al., 2011 [83]	Straight metalworking fluids (PAHs)	Cohort	Autoworkers, USA	Melanoma	Significant increase	No	Co-exposures
Partanen and Boffetta 1994 [86]	PAHs (asphalt/tar)	Meta-analysis	Asphalt workers and roofers	Skin cancer	Increased risk	Variable	Occupational mix
Dennis et al., 2010 [71]	Carbaryl, parathion, maneb/mancozeb	Cohort	AHS pesticide applicators	Melanoma	OR 1.7–2.4 with dose–response	Yes	Applicator population; generalisability
Fortes et al., 2016 [69]	Pesticides (mixed; fungicides, herbicides)	Pooled case–control	Italian and Brazilian populations	Melanoma	OR 7.40 (≥10 years); fungicides OR 3.08	Partial	Recall bias; mixed pesticides
Stanganelli et al., 2020 [78]	Herbicides	Meta-analysis	Multiple studies	Melanoma	SRR 1.85 (1.01–3.36)	Variable	Heterogeneity
Tseng et al., 1968 [113]	Inorganic arsenic (drinking water)	Ecologic	Endemic area, Taiwan	NMSC prevalence	Dose–response	No	Ecologic design
Gilbert-Diamond et al., 2013 [118]	Urinary arsenic species	Case–control	New Hampshire, USA	SCC	Significant association	Yes	
Karagas et al., 2001 [119]	Toenail arsenic	Case–control	US population-based	BCC, SCC	Significant for SCC	Yes	
He et al., 2025 [122]	Arsenic (mixed)	Systematic review/meta-analysis	Multiple studies	Melanoma and NMSC	Significant for NMSC	Variable	Heterogeneity
Bedaiwi et al., 2022 [123]	Urinary arsenic (NHANES)	Cross-sectional	US adults	Melanoma	Significant association	No	Cross-sectional design

## 15. Discussion

This narrative review examined the evidence for an association between exposure to selected EDCs and skin cancer. The overall evidence varies considerably by chemical class, cancer type, and exposure level. A consistent finding is that high chronic occupational exposure—rather than background environmental exposure—is the most strongly and consistently associated with skin cancer risk.

Arsenic and certain PAHs (particularly tar/pitch and straight metalworking fluid exposures) have the most consistent and dose-dependent evidence for skin carcinogenicity, supported by in vitro, animal, and human data. For pesticides, the evidence is strongest for specific compounds (carbaryl, parathion, maneb/mancozeb) and for prolonged exposures (>10 years), supported by dose–response data from large prospective cohorts such as the AHS. PCBs have moderate and inconsistent evidence, with some cohort and case–control studies showing increased melanoma risk but meta-analyses overall failing to demonstrate a significant association. Evidence for TCDD, PFAS, PBDEs, phthalates, parabens, VCM, and DES is limited or insufficient for skin cancer specifically.

A critical observation is that, while endocrine-disrupting activity and carcinogenicity are often discussed in parallel, there is limited direct evidence that interference with hormone signalling mechanistically mediates skin cancer development for most compounds. AhR-mediated signalling represents the most plausible mechanistic link between the endocrine and carcinogenic properties of dioxin-like PCBs, TCDD, and PAHs. For arsenic and pesticides, the primary carcinogenic mechanisms (DNA repair inhibition, oxidative stress) do not involve endocrine disruption in a direct sense.

UV radiation is the principal established environmental risk factor for skin cancer and a major potential confounder in all occupational studies. Many occupational populations reviewed had substantially higher UV exposure than the control populations. This confounding was inadequately addressed in the majority of studies. Future studies should systematically collect and adjust for UV exposure, skin phototypes, and socioeconomic factors.

## 16. Conclusions

High chronic exposure to specific EDCs—especially arsenic, certain PAHs, and certain pesticides (particularly carbaryl, parathion, and maneb/mancozeb)—may increase skin cancer risk. The evidence is strongest for NMSC with arsenic exposure and for SCC/NMSC with high occupational PAH exposure. The evidence for melanoma is more heterogeneous. Further research is required, particularly prospective studies with careful exposure assessment and adequate UV adjustment. For most chemicals reviewed, direct evidence that endocrine disruption specifically mediates skin carcinogenesis is lacking; an association with carcinogenicity and an association with endocrine disruption does not necessarily imply that one causes the other.

## Data Availability

No new data were created or analysed in this study. Data sharing is not applicable to this article.
